# Phenotypic diversity and selection in biofortified cassava germplasm for yield and quality root traits

**DOI:** 10.1007/s10681-022-03125-6

**Published:** 2022-11-17

**Authors:** Ravena Rocha Bessa de Carvalho, Massaine Bandeira e Sousa, Luciana Alves de Oliveira, Eder Jorge de Oliveira

**Affiliations:** 1 Centro de Ciências Agrárias, Ambientais e Biológicas, Universidade Federal do Recôncavo da Bahia, Cruz das Almas, BA 44380-000 Brazil; 2 Embrapa Mandioca e Fruticultura, Nugene, Cruz das Almas, BA 44380-000 Brazil

**Keywords:** Carotenoids, Breeding, *Manihot esculenta* Crantz, Root quality

## Abstract

Increasing carotenoid content and improving other root quality traits has been the focus of cassava biofortification. This study aimed to (i) evaluate the genetic variability for total carotenoid content (TCC), as well as for root yield and root quality attributes; (ii) estimate potentially useful correlations for selection; and (iii) select parents for breeding and estimate the genetic gain. Data from 2011 to 2020 of 265 cassava genotypes with cream and yellow roots were analyzed for dry matter content (DMC), shoot yield, fresh root yield (FRY), dry root yield (DRY), harvest index, average number of roots per plant, starch content, root pulp color, cyanogenic compounds, and TCC. The best linear unbiased predictions showed great phenotypic variation for all traits. Six distinct groups were formed for productive characteristics of root quality, mainly TCC, DMC and FRY. Only TCC showed high broad-sense heritability (${h}^{2}$= 0.72), while the other traits had low to medium magnitude (0.21 ≤ ${h}^{2}$ ≤ 0.60). TCC was strongly correlated with pulp color (r = 0.70), but null significance for DMC. The network analysis identified a clear separation between the agronomic and quality attributes of cassava roots. The selection of the 30 genotypes for recombination in the breeding program has the potential to raise TCC by 27.05% and reduce the cyanogenic compounds content by 23.03%, in addition to increasing FRY and DRY by 22.72% and 22.95%, respectively. This is the first consolidated study on the potential of germplasm for the development biofortified cassava cultivars in Brazil.

## Introduction

Cassava (*Manihot esculenta* Crantz) is a starchy root crop widely cultivated in Southeast Asia, Latin America, the Caribbean and Sub-Saharan Africa for human and animal feed and as a raw material for biofuels and various other industrialized products with a wide range of uses (Howeler et al. 2013). Nigeria is the world′s largest producer of cassava root, followed by the Democratic Republic of Congo, Thailand, and Ghana, producing 59.19, 40.05, 31.07, and 22.44 million tons of roots, respectively, in 2019 (Food and Agriculture Organization of the United Nations 2019). Brazil stands out as the fifth largest producer globally, harvesting 17.49 million tons from approximately 1.19 million hectares, giving a national root yield of 14.70 t ha^− 1^ (Food and Agriculture Organization of the United Nations 2019). However, there are large variations in the national root yield of cassava in Brazil, varying from 9.8 t ha^− 1^ in the northeast to 21.7 t ha^− 1^ in the mid-south (Instituto Brasileiro de Geografia e Estatística 2019).

Cassava roots are an excellent source of calories due to the starch accumulation (Montagnac et al. 2009). Indeed, starch is the main commercially exploited constituent of cassava, whose uses in the food, paper, cellulose, textile, and biofuel industries, among others, are widely recognized (Balagopalan 2002; Food and Agriculture Organization of the United Nations 2019). Cassava is the second most important source of starch in the world (Stapleton 2012). In addition to starch, cassava root contains water, fiber, protein, fat, mineral elements (Zn, Fe, N, Ca, P, K and Mg) and vitamins (B1, B2, B3, C and β-carotene) (Sayre et al. 2011; Parmar et al. 2017).

Cassava has high environmental resilience and is able to guarantee minimum yields even when subjected to various environmental stresses, such as water deficits, cultivation in soil of low natural fertility, and management with low use of agricultural inputs (Burns et al. 2010; Jarvis et al. 2012). It is an easily adaptable crop to scenarios associated with future climate change, thus having a high potential to improve food security in economically less favored regions (Food and Agriculture Organization of the United Nations 2019). Cassava breeding programs seek to identify and select genotypes with desirable traits such as higher root and starch yield, increased resistance to pests and diseases (Oliveira et al. 2015a), lower toxicity (Parmar et al. 2017), improved tolerance to water deficit (Koundinya et al. 2018), and better nutritional content (Esuma et al. 2012; Ceballos et al. 2013).

The biofortification of staple crops is a global initiative aimed at ensuring food security for the population, providing essential nutrients for human development (Mayer et al. 2008; Saltzman et al. 2017; Bouis and Saltzman 2017; Alamu et al. 2019). The cassava biofortification process aims to increase the carotenoids content through conventional breeding techniques, taking advantage of the natural genetic variability of the species (Esuma et al. 2012; Ceballos et al. 2013), as well as through genetic transformation (Welsch et al. 2010; Failla et al. 2012).

Carotenoids play a fundamental role in human health (Cuevas et al. 2010), as β-carotene—the most abundant carotenoid in cassava (Ceballos et al. 2017)—acts as a vitamin A precursor with antioxidant properties (Shete and Quadro 2013). Therefore, cassava is a strong candidate species to act as a source of carotenoids, especially in regions where its consumption is high due to traditional cultural factors as well as the difficulty of cultivating other species, as seen in the semi-arid region in northeastern Brazil.

Most of the cassava cultivars released in Brazil before 2002 had a white pulp color (Fukuda et al. 2002). The report of carotenoid content in cassava from improved clones and landraces varied from 1.02 to 10.40 µg g^− 1^ in fresh roots (Chávez et al. 2005). In recent years, new biofortified cultivars launched in Brazil exhibited total carotenoid contents (TCC) ranging between 3.3 and 8.7 µg g^− 1^ of β-carotene (Fukuda et al. 2005, 2009; Fukuda and Pereira 2005). However, recent studies have shown a variation between 0.20 and 30.84 µg g^− 1^ in cassava germplasm (Sánchez et al. 2014; Rabbi et al. 2017). The presence of genetic variability for TCC has allowed the development of biofortified germplasm using conventional breeding strategies to overcome vitamin A deficiency in sub-Saharan Africa (Sayre et al. 2011; Njoku et al. 2014; Talsma et al. 2016) and in Latin America (Meenakshi et al. 2010; Ceballos et al. 2013).

The possibility of combining biofortified cultivars with desirable culinary traits and high agronomic performance has demanded huge attention from breeders (Belalcazar et al. 2016; Fuhrmann et al. 2019; Parkes et al. 2020). However, the cassava biofortification program should focus on the development of fresh consumption cultivars (sweet cassava), considering that the retention of carotenoids in cooked root samples is between 95 and 100% (Taleon et al. 2019). In this case, in addition to the high content of carotenoids, the biofortified sweet cultivars must have a low cyanogenic compounds content and high dry matter content in the roots, associated with high agronomic performance.

The bitter cassava cultivars contain high concentrations of cyanogenic compounds (linamarina and lotaustralin) throughout the plant, which are effectively reduced after various processing and fermentation methods (Parmar et al. 2017). The genotypes can be classified according to the concentration of cyanogenic glycosides in the roots as bitter cassava (≥ 100 mg cyanogenic compounds kg^− 1^) or sweet cassava (≤ 100 mg cyanogenic compounds kg^− 1^) (Mckey et al. 2010).

The dry matter content in the roots can range from 8.4 to 52.5% (Sánchez et al. 2014; Oliveira et al. 2015b, 2017; Rabbi et al. 2017), having wide natural variation for improvement. However, several reports from germplasm panels (containing improved clones and local cultivars) in Africa have demonstrated the existence of negative correlations (− 0.22 to − 0.59) between carotenoid content and dry matter content in the roots (Akinwale et al. 2010; Esuma et al. 2012; Njoku et al. 2015; Rabbi et al. 2017). Recently, researchers analyzed a panel of 672 African cassava clones and identified two loci on chromosome 1 at 24.1 and 30.5 Mbp segments, associated with cassava root color (highly correlated with carotenoid content), and a single locus for dry matter content in the region close to 24.1 Mbp on chromosome 1 (Rabbi et al. 2017). These authors therefore reported the existence of a physical link between these two traits in the African cassava germplasm.

Ensuring the adoption of biofortified cassava cultivars depends on the generation of progenies that allow a simultaneous increase in the carotenoids content and agronomic traits that are essential to guarantee the roots′ commercialization (high dry matter content, low cyanogenic compounds content, and adequate root size and shape) and at the same time their profitability (high root yield, pest and disease resistance, and adaptation to mechanized planting systems).

One of the key steps to including new clones as parents in crossing blocks is the characterization of their yield potential, disease resistance, suitability to the target crop management, and above all, good root quality. This step allows the identification of parent combinations that can maximize the recombinations to generate segregating progenies with high genetic variability while simultaneously allowing the introgression of desirable genes (Oliveira et al. 2014). However, few studies have been dedicated to a broad characterization of Brazilian cassava germplasm for all these traits associated with the acceptance of biofortified cultivars. Therefore, this study aimed to evaluate the phenotypic variability for traits associated with root yield and root quality of biofortified cassava genotypes, and further to select the most promising clones for breeding. The correlations between the different traits evaluated and the potential genetic gain with the selection of the best clones for crossing blocks were also discussed.

## Materials and methods

### Field trials for data collection

In this study, data from the cassava breeding program of Embrapa Mandioca e Fruticultura was used. A total of 21 trials were conducted in the Bahia cities of Cruz das Almas, Laje and Valença from 2011 to 2020. Detailed information about each trial′s soil type, coordinates and years of evaluation are presented in Table 1. According to the Köppen classification, the climate of the regions is Af, indicating a warm, humid, tropical climate, with average annual precipitation ranging from 1200 to 1500 mm, higher rain incidence in the period between March and July, an average annual temperature of 24.5ºC, and an average relative humidity of approximately 80%.

**Table 1:** Location and characterization of the field trials used for cassava germplasm evaluation

Code	Year	Location	City	Altitude	Coordinates	Soil type*
AgroV1	2011	Embrapa	Cruz das Almas	215	12°40′36.7"S 39°05′08.0"W	YelOxi
AgroV2	2011	Embrapa	Cruz das Almas	210	12°40′32.6"S 39°05′13.7"W	YelOxi
BAG-C	2012	Embrapa	Cruz das Almas	200	12°40′47.4"S 39°05′00.2"W	YelOxi
AgroV1	2013	Bahiamido	Laje	180	13°06′38.4"S 39°16′20.4"W	RedOxi
AgroV1	2013	UFRB	Cruz das Almas	210	12°39′25.9"S 39°04′58.8"W	YelOxi
BAG-C	2013	Embrapa	Cruz das Almas	217	12°40′22.8"S 39°05′06.1"W	YelOxi
BAG-1.2	2014	Embrapa	Cruz das Almas	216	12°40′22.8"S 39°05′01.5"W	YelOxi
BAG-1.1	2014	Embrapa	Cruz das Almas	216	12°40′22.8"S 39°05′01.5"W	YelOxi
BAG-1	2014	Bahiamido	Laje	180	13°06′39.6"S 39°16′17.6"W	YelOxi
BAG-2	2014	Bahiamido	Laje	175	13°06′35.6"S 39°16′19.3"W	YelOxi
BAG-3	2014	Bahiamido	Laje	180	13°06′39.6"S 39°16′17.6"W	YelOxi
BAG-1.1	2015	Embrapa	Cruz das Almas	216	12°40′22.8"S 39°05′01.5"W	YelOxi
BAG-1	2015	Bahiamido	Laje	196	13°06′38.5"S 39°16′49.0"W	YelOxi
BAG-2	2015	Bahiamido	Valença	40	13°15′33.5"S 39°14′12.8"W	RedYelOxi
BAG-2	2015	UFRB	Cruz das Almas	210	12°39′16.4"S 39°04′53.4"W	YelOxi
BAG-3	2015	Bahiamido	Laje	196	13°06′38.5"S 39°16′49.0"W	YelOxi
BAG-4	2015	Embrapa	Cruz das Almas	216	12°40′19.5"S 39°05′02.5"W	YelOxi
BAG-1.1	2018	UFRB	Cruz das Almas	223	12°39′51.4"S 39°04′15.7"W	YelOxi
BAG-1.1	2019	UFRB	Cruz das Almas	223	12°39′43.5"S 39°04′12.0"W	YelOxi
BAG-1.1	2020	UFRB	Cruz das Almas	225	12°39′49.2"S 39°03′58.1"W	YelOxi
BAG-1.2	2020	UFRB	Cruz das Almas	225	12°39′49.2"S 39°03′58.1"W	YelOxi

*YelOxi = Yellow Oxisol, RedOxi = Red Yellow Oxisol, RedYelOxi = Red Yellow Oxisol

The field trials were performed in an augmented block design, with plots composed of two lines with eight to ten plants each (giving a total of 16–20 plants per plot), spaced 0.90 m between rows and 0.80 m between plants. Between 4 and 34 improved Embrapa cultivars and local cultivars were planted as common checks in the different augmented blocks (ranging from 5 to 22). We adopted the conventional crop system used in the region to prepare the soil (first plowing, next harrowing, then opening the planting furrows using the cassava planter, without soil cover discs). We used stakes of approximately 16–18 cm in length.

All field trials were conducted under rainfed conditions (without complementary irrigation), following the local crop management, in accordance with the recommendations of (Souza et al. 2006). Planting was conducted during the rainy season in the region (May to August) to ensure the minimum moisture in the soil necessary for germination and crop establishment.

### Cassava germplasm panel

The cassava panel analyzed consisted primarily of genotypes with cream to yellow pulp roots, considering that the yellow color of cassava roots is directly associated with their carotenoids content (Esuma et al. 2016). Therefore, a panel with 265 cassava accessions consisting of improved and local (non-improved) cultivars was selected (Supplementary Table S1). Most of this germplasm originated in Colombia (6%) and Brazil (3%, 34%, 37%, 4%, and 6% in the mid-west, north, northeast, south, and southeast regions, respectively, in addition to another 9% of unknown origin).

### Agronomic data collection

The harvest stage of the trials was manually performed 10–12 months after planting. The following characteristics were assessed:

1. dry matter content (DMC) in the roots based on the gravimetric method (DMC.Grav) in %: approximately 5 kg of roots were cleaned to remove excess soil and their ends were cut off. Then, the weight of the roots in air and water were obtained and the DMC.Grav was calculated based on the following formula: ${\text{DMC~}} = \,158.3 \times \,\frac{{{\text{Weight~in~air}}}}{{{\text{Weight~in~air~}} - {\text{~Weight~in~water}}}} - 142$, according to (Kawano et al. 1987);
2. dry matter content in the roots based on sample oven drying (DMC.OD) in %: around 3 to 5 roots of different plants were selected and cleaned to remove excess soil. Then, approximately 200 g of roots from different root positions were crushed to facilitate drying in an oven at 90º C until they reached a constant weight (generally achieved within 48 h). The DMC.OD was determined by the formula: DMC.OD$\, = \,100\, - {\text{~humidity~}}\left( {\text{\% }} \right)$;
3. shoot yield (ShY): obtained by harvesting and removing the roots of the plants, weighing only the shoot parts of all plants in the plot, and adjusted to t ha^− 1^;
4. fresh root yield (FRY): obtained by weighing all the roots of the plot, using a digital scale, and converted to t ha^− 1^;
5. harvest index (HI) in %: the ratio between root and shoot weight, calculated according to the following formula: ${\text{HI~}} = \,\frac{{FRY}}{{FRY + ShY}}\, \times 100$;
6. dry root yield (DRY) in t ha^− 1^: the product of FRY and DMC.Grav;
7. average number of roots per plant (NRP): obtained by counting all the roots in the plot divided by the number of plants harvested;
8. starch content in roots (StC) in %: starch from approximately 1 kg of roots from different plants in the plot was extracted according to Vasconcelos et al. (2017);
9. root pulp color (PulpColor): classified as 1 = white roots, 2 = cream roots and 3 = yellow roots;
10. cyanogenic compounds content (HCN): obtained by the picrate method (Fukuda et al. 2010).

### Total carotenoid content analysis

For the analysis of TCC, DMC.OD and StC, the samples were taken to the Laboratory of Cassava Crop Management during the early morning hours. All roots were washed, peeled, and cut into small pieces before starting the analysis. TCC was only assessed in 2019 and 2020.

For TCC, the roots were always manipulated under low light restrictions. Two samples containing 10 g, 15 or 25 g of ground root (depending on the intensity of the pulp color) and a reserve sample of 60 g were collected in glass jars with lids wrapped in aluminum foil. TCC was quantified as described in the HarvestPlus Handbook for Carotenoid Analysis (Rodriguez-Amaya and Kimura 2004). First, the pigments were extracted from the crushed cassava sample with approximately 50 mL of acetone, through grinding using the Ultra Turrax homogenizer.

The mixture containing the sample and acetone was then filtered through a Buchner funnel with the aid of a vacuum pump, and the residue retained in the funnel was washed with acetone until it did not show any color. The extract containing only the pigment and acetone, reserved in the suction flask, was transferred to a separating funnel containing petroleum ether (a variable amount depending on the color of the sample), and approximately 250 mL of saline solution was slowly added to induce a separation of phases. The aqueous phase was discarded and the saline washing procedure was repeated five times until only petroleum ether and pigment remained. This new extract was filtered through a funnel with glass wool and anhydrous sodium sulfate into an amber volumetric flask and its volume was supplemented with petroleum ether. An aliquot of each sample was used to determine the TCC using a spectrophotometer (UV-Vis Thermo Scientific, Genesis 10 S model) adjusted for absorbance at 450 nm.

The TCC was calculated using the following formula: ${\text{TCC}}\left( {ug.g^{{ - 1}} } \right)\, = \,\frac{{A \times V\left( {mL} \right) \times 10^{4} }}{{A_{{1cm}}^{{1\% }}  \times P\left( g \right)}}$, where $A$ is the absorbance, $V\left(mL\right)$ is the total extract volume in milliliters, $P\left(g\right)$is the sample weight in grams, and ${A}_{1 cm}^{1\%}$ is equal to the extinction coefficient of β-carotene in petroleum ether (2592).

### Data analysis

A mixed model approach was used for data analysis, following the formula ${Y}_{ijk}= \mu +{E}_{i}+{B}_{\left(i\right)j}+{G}_{k}+{GE}_{ik}+{\varepsilon}_{ijk}$, where ${Y}_{ijk}$ is the observed value of genotype *k* in block *j* from environment *i*; *µ* is the general constant of the experiment; ${E}_{i}$ is the (random) effect of the environment *i*, with *i* = 1, 2, … *n*; ${B}_{\left(i\right)j}$ is the block effect *j*, with *j* = 1, 2, … *n*, within environment *i*; and ${G}_{k}={T}_{k{\prime }}+{T}_{\left(j\right)k}$, where ${G}_{k}$ is the genotype effect, ${T}_{k{\prime }}$ is the fixed effect of the common treatment *k′*= 1, 2, … *n*, and ${T}_{\left(j\right)k}$ is the random effect of regular treatment *k* within block *j*. Further, note that ${T}_{\left(i\right)k}$ ~ NID (0,${\sigma }_{T\left(j\right)k}^{2}$); ${GE}_{ik}$ is the (random) effect of the environment *i* interaction with genotype *k*; ${GE}_{ik}$ ~ NID (0,${\sigma }_{GA\left(j\right)k}^{2})$; and ${\varepsilon}_{ijk}$ is the random effect of the experimental error ~ NID (0, ${\sigma }^{2}$).

The model effects were estimated with the lme4 package (Bates et al. 2015) of R software version 4.03 (R Core Team 2021). The variance components were estimated by the restricted maximum likelihood and then the best linear unbiased predictors (BLUPs) were obtained for the random effects. The significance of each model effect was tested based on deviance analysis, according to the likelihood ratio test (LRT), using the χ² distribution at 1% probability.

The broad sense heritability was estimated by ${h}^{2}=\frac{{\sigma}_{g}^{2}}{{\sigma}_{g}^{2}+{{\sigma}_{ga}^{2}+}_{e}^{2}}$, while the broad sense heritability by plot (clonal mean heritability) in the mean of genotypes was obtained by the formula ${h}_{m}^{2}=\frac{{\sigma}_{g}^{2}}{{\sigma}_{g}^{2}+\frac{{\sigma}_{ga}^{2}}{e}+\frac{{\sigma}_{e}^{2}}{re}}$, where ${\sigma}_{g}^{2}$ is the genotype variance, ${\sigma}_{ga}^{2}$ is the genotype × trials interaction variance, ${\sigma}_{e}^{2}$ is the error variance, $e$ is the number of trials, and *r* is the product of the number of replicates adjusted by the number of trials.

The correlations between yield traits, root quality, and carotenoid content were estimated using the Pearson correlation test, for which the hypothesis of correlation equal to zero was analyzed by the *t* test with n-2 degrees (p < 0.05), using the corrplot package (Wei and Simko 2021) of the R software version 4.03 (R Core Team 2021). The hierarchical clustered correlogram is designed to show the magnitude and direction of correlations. In addition, the network correlation was performed using the qgraph package (Epskamp 2012) of the R software version 4.03 (R Core Team 2021).

The number of cassava clusters based on several attributes associated with root quality and agronomic aspects were determined based on successive K-means and increasing number of groups (k ranging from 2 to 15) after transforming the dataset by principal component analysis using the find.clusters function of the adegenet package (Jombart et al. 2010) in version R 4.0.3. The number of clusters was compared using the Bayesian Information Criterion (BIC), and the most suitable clustering solution was identified as that with the lowest BIC. After determining the most adequate number of groups to represent the germplasm diversity, the dendrogram was constructed using the circlize package (Gu et al. 2014) in version R 4.0.3.

Boxplots obtained using the ggstatsplot package (Patil 2018) in version R 4.0.3 were used to visualize the distribution and existence of differences between groups for agronomic and root quality attributes. Thirty cassava genotypes were selected for recombination and generation of improved biofortified progenies of sweet cassava (with high carotenoids content and low HCN), associated with high agronomic performance. The following selection index was used based on the sum of ranks (Mulamba and Mock 1978) and predefined weights: $SI = \left( {{\text{DMC}}.{\text{Grav}} \times 5} \right) + \left( {{\text{DMC}}.{\text{OD}} \times 5} \right) + \left( {{\text{TCC}} \times 30} \right) + \left( {{\text{FRY}} \times 10} \right) + \left( {{\text{ShY}} \times 5} \right) + \left( {{\text{HI}} \times 5} \right) + \left( {{\text{DRY}} \times 10} \right) + \left( {{\text{NRP}} \times 10} \right) + \left( {{\text{StC}} \times 5} \right) + \left( {{\text{PulpColor}} \times 30} \right) + \left( {{\text{HCN}} \times  - 30} \right)$, which refers to the sum of the BLUPs of each trait multiplied by their respective economic weights. We also calculated the genetic gain by using the formula $G={h}_{m}^{2}S$, where, $G$ is the genetic gain and $S$ is the BLUP deviation of the selected genotypes from the population mean, according to Schmidt et al. (2019).

## Results

### Variation and genetic parameters for carotenoid content, yield, and root quality traits

**Fig. 1:**
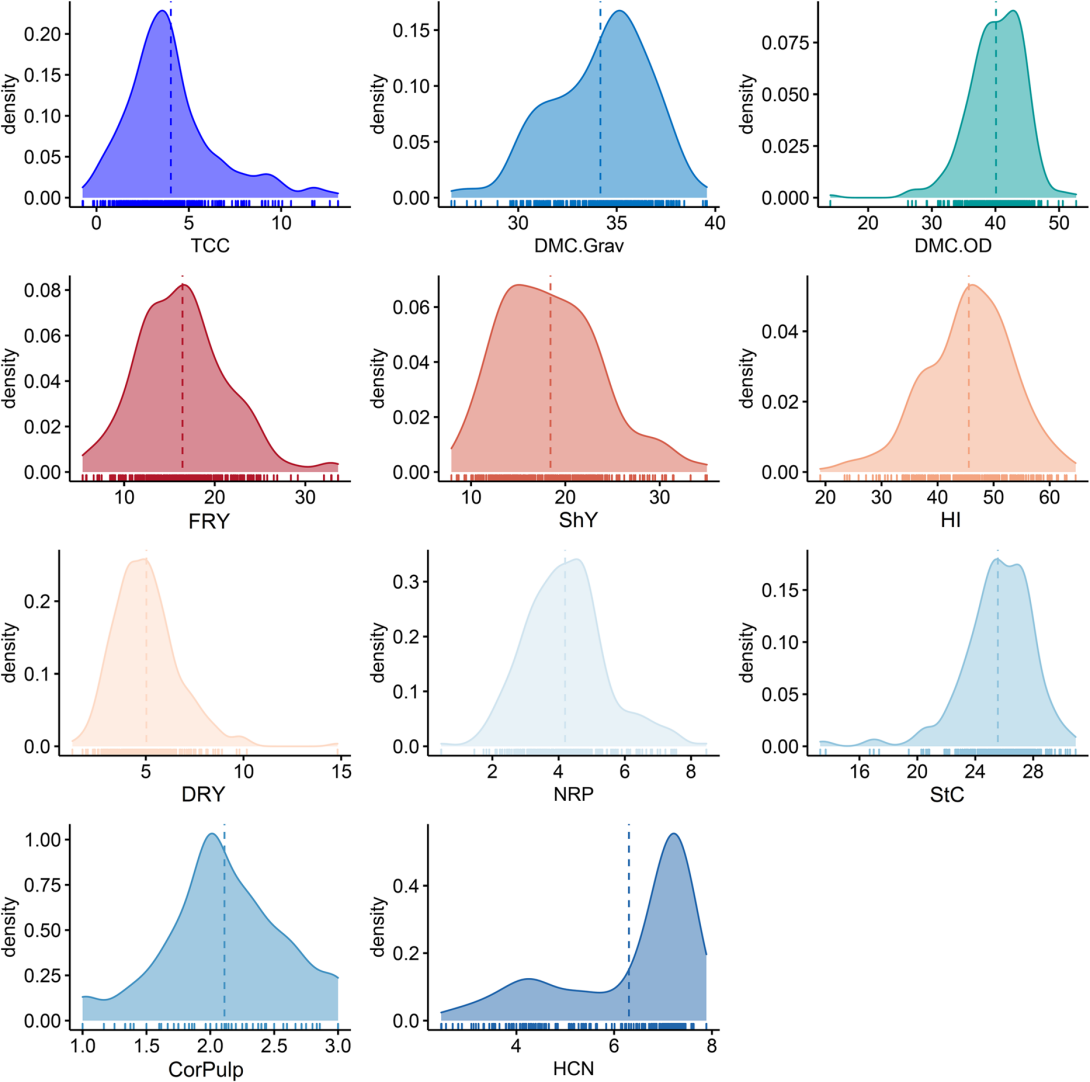
Distribution of the best linear unbiased prediction (BLUP) added to the overall mean, for several attributes associated with root quality and root yield of yellow cassava genotypes. TCC: total carotenoid content; DMC.Grav: dry matter content obtained by the gravimetric method; DMC.OD: dry matter content measured by the oven dry method; FRY: fresh root yield; ShY: shoot yield; HI: harvest index; DRY: dry root yield; NRP: number of roots; StC: starch content; CorPulp: pulp color of the roots; HCN: cyanogenic compounds content

The distribution of BLUPs added to the intercept (henceforth simply called BLUPs) for all traits is presented in Fig. 1. The range of BLUPs variation for TCC was reasonably high (0.075–13.08 µg g^− 1^), with an average of 4.09 µg g^− 1^. The same was observed for other traits associated with root quality such as StC (range 13.29–30.93% and mean 25.36%), PulpColor (score range 1–3 with mean 2.11) and HCN (score range 2.46–7.97 with mean 6.3). This demonstrates that most of the genotypes evaluated exhibited cream-colored pulp and still had a high HCN. This statement can be evidenced by the greater distribution of genotypes to the right of the density graph, that is, exhibiting high HCN. For the other traits, there is a distribution close to normality.

Regarding the DMC of the roots, the amplitude of the data obtained by the gravimetric method (26.61–39.57% with an average of 34.22%) was much smaller than that of the oven dry method (14.07–52.67% with an average of 39.99%). Although the gravimetric method provides a simpler and faster method to obtain the DMC, the DMC.Grav value is an estimate obtained indirectly from the regression proposed by Kawano et al. (1987), and therefore there may be errors implicit in this methodology. Conversely, DMC.OD offers a direct analysis to obtain the DMC of cassava.

The phenotypic variation in yellow pulp cassava germplasm also showed wide variation for several agronomic traits, including FRY (ranging from 5.41 to 33.62 t ha^− 1^ with an average of 16.49 t ha^− 1^), ShY (7.92–35.00 t ha^− 1^ and average of 18.57 t ha^− 1^), HI (19.05–64.64% and average of 45.40%), DRY (1.24–14.81 t ha^− 1^ and average of 5.06 t ha^− 1^), and NRP (0.45–8.46 with an average of 4.15 roots per plant). Therefore, this biofortified cassava germplasm panel has high potential for genotype selection for the recombination of important traits for the sweet cassava breeding program, such as the high TCC and DMC and low HCN.

The deviance analysis indicated the significance of genotypes′ effects for all traits (Table 2), reinforcing previous findings related to the high carotenoids content and other attributes associated with cassava root quality and yield. Furthermore, the effect of the genotype × trial interaction was significant for all traits except TCC, PulpColor, DMC.OD, and StC.

**Table 2:** Summary of the phenotypic variation, estimates of variance components and heritability for total carotenoid content (TCC), dry matter content by the gravimetric method (DMC.Grav) and oven dry method (DMC.OD), fresh (FRY) and dry root yield (DRY), shoot yield (ShY), harvest index (HI), number of roots (NRP), starch content (StC), cyanogenic compounds content (HCN) and pulp color of the roots (PulpColor).

Traits	Mean	${h}^{2}$	${h}_{m}^{2}$	${\sigma }_{g}^{2}$	${\sigma }_{ge}^{2}$	${\sigma }_{e}^{2}$	${\chi}_{g}^{2}$	${\chi}_{ge}^{2}$
TCC	4.09	0.72	1.00	5.44	0.00	2.16	100.36*	0.00
DMC.Grav	34.22	0.45	0.92	5.26	3.87	2.59	852.71*	288.62*
DMC.DO	39.99	0.54	0.98	13.15	1.68	9.44	48.74*	0.02
PTR	16.49	0.21	0.83	17.28	28.06	37.56	281.36*	209.28*
PPA	18.57	0.22	0.81	19.12	35.14	32.50	287.30*	305.41*
IC	45.40	0.32	0.89	49.42	46.83	56.40	532.64*	151.22*
PROD.Dry	5.06	0.21	0.80	1.78	3.49	3.22	203.33*	227.16*
NRP	4.15	0.32	0.89	0.81	0.80	0.89	41.30*	31.24*
AMD	25.36	0.48	1.00	5.17	0.00	5.59	15.26*	0.00
HCN	6.80	0.60	0.96	1.60	0.53	0.52	128.22*	150.61*
CorPolpa	2.11	0.42	0.99	0.18	0.00	0.25	31.61*	0.00

${h}^{2}$= broad-sense heritability; ${h}_{m}^{2}$ = broad-sense heritability per plot of genotype means; ${\sigma }_{g}^{2}$= genotype variance; ${\sigma }_{ge}^{2}$ = variance of the genotype × trial interaction; ${\sigma }_{e}^{2}$ = error variance; ${\chi}_{g}^{2}$= chi-square of genotype effect; ${\chi}_{ge}^{2}$= chi-square of the effect of the genotype × trial interaction; * = significant at *p* < 0.01

Regarding the genetic parameters, only TCC showed high broad-sense heritability (${h}^{2}$= 0.72), while the traits PulpColor, DMC.Grav, StC, DMC.OD, and HCN had ${h}^{2}$of medium magnitude (0.42, 0.45, 0.48, 0.54, and 0.60, respectively). In contrast, the yield traits were those with the lowest heritability (${h}^{2}$= 0.32 for NRP and HI, and 0.21 $\le {h}^{2}\le$ 0.22 for FRY, DRY and ShY). However, the broad-sense heritability per plot was quite high for all traits, especially FRY, DRY, ShY, NRP and HI (0.80 $\le {h}_{m}^{2}\le$0.89).

### Correlations between traits

**Fig. 2:**
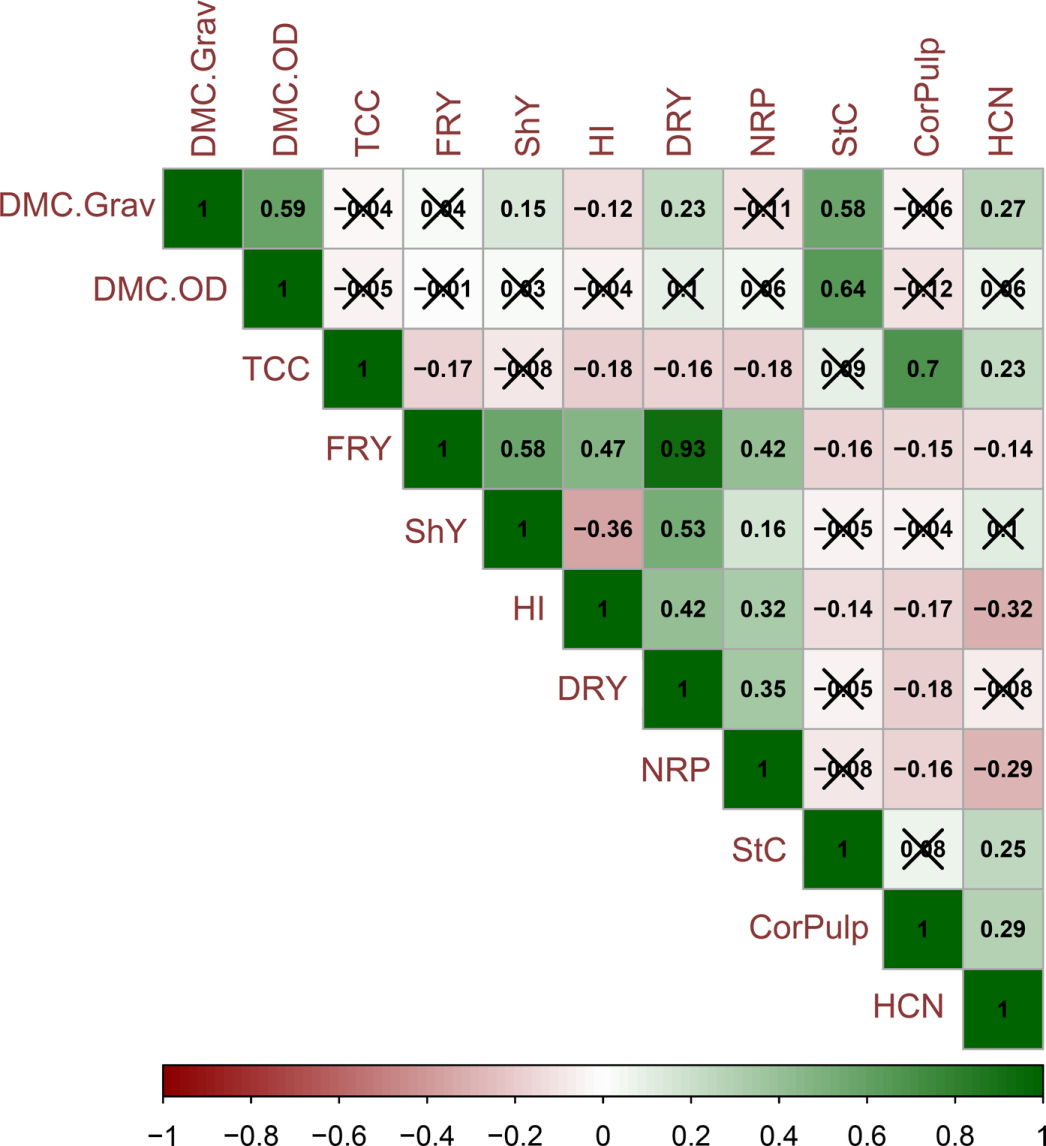
Pearson′s correlogram between agronomic and root quality traits in cassava evaluated in biofortified cassava genotypes. TCC: total carotenoid content; DMC.Grav and DMC.OD: dry matter content by the gravimetric method and oven dry method, respectively; FRY: fresh root yield; ShY: shoot yield; HI: harvest index; DRY: dry root yield; NRP: number of roots; StC: starch content; CorPulp: pulp color of the roots; HCN: cyanogenic compounds content. Correlations with “x” are not significant (p > 0.05)

Figure 2 shows Pearson′s correlations, which indicated strong positive correlations between TCC × PulpColor (r = 0.70) and FRY × DRY (r = 0.93) and positive correlations of medium magnitude between DMC.Grav *versus* DMC.OD (r = 0.59) and StC (r = 0.58), DMC.OD × StC (r = 0.64), FRY × ShY (r = 0.58), and ShY × DRY (r = 0.53). In addition, positive correlations of low magnitude that were significant were identified between FRY *versus* HI (r = 0.47) and NRP (r = 0.42), HI *versus* PROD,Dry (r = 0.42) and NRP (r = 0.32), PROD,Dry × NRP (r = 0.35) and PulpColor × HCN (r = 0.29). The negative correlations observed were significant but of low magnitude, including those between ShY × HI (r = − 0.36), HI × HCN (r = − 0.32) and NRP × HCN (r = − 0.29). Finally, the correlations between TCC *versus* DMC.Grav and DMC.OD were negative (r = − 0.04 and − 0.05, respectively), but practically null and without significance.

**Fig. 3:**
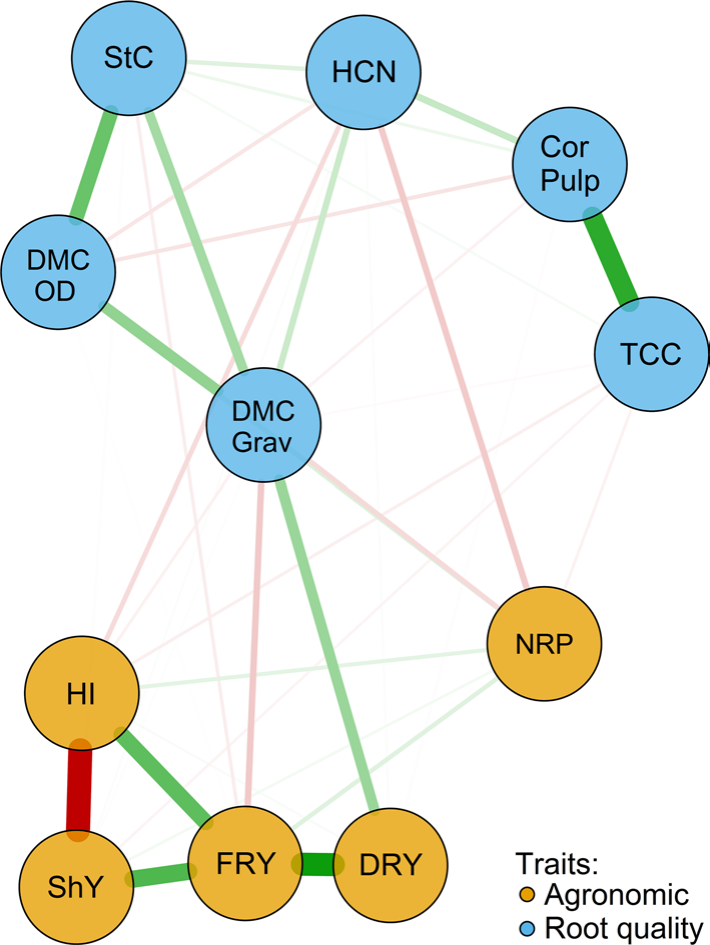
Regularized network of partial correlations between agronomic and root quality traits evaluated in biofortified cassava genotypes. The thickness of the lines represents the correlations, while the green and red colors represent positive and negative correlations, respectively. TCC: total carotenoid content; DMC.Grav: dry matter content evaluated by the gravimetric method; DMC.OD: dry matter content measured by the oven dry method; FRY: fresh root yield; ShY: shoot yield; HI: harvest index; DRY: dry root yield; NRP: number of roots; StC: starch content; CorPulp: pulp color of the roots; HCN: cyanogenic compounds content

The network based on all pairs of significant correlations between agronomic and cassava root quality traits is shown in Fig. 3. As expected, this network exhibited similar trends to the correlogram (Fig. 2). Furthermore, this network identified a clear separation between the agronomic and root quality traits. Additionally, regardless of the direction of the correlation, there was a clear, strong relationship between the agronomic traits HI, ShY, FRY and DRY, while the NRP trait had a weaker positive association with the other agronomic attributes and a negative one with the root quality traits. Among the agronomic traits, HI and FRY were those with the greatest number of connections with the others, while ShY and DRY exhibited the smallest number of connections.

For the indicators of root quality, two subgroups of traits formed, with DMC.Grav, DMC.OD and StC in one subgroup and HCN, TCC and PulpColor in the other. On the one hand, despite the weak correlations (both negative and positive), HCN and DMC.Grav had a greater number of connections with other attributes. On the other hand, TCC was basically related to PulpColor and some yield traits via weak correlations.

### Diversity pattern of biofortified cassava clones

**Fig. 4:**
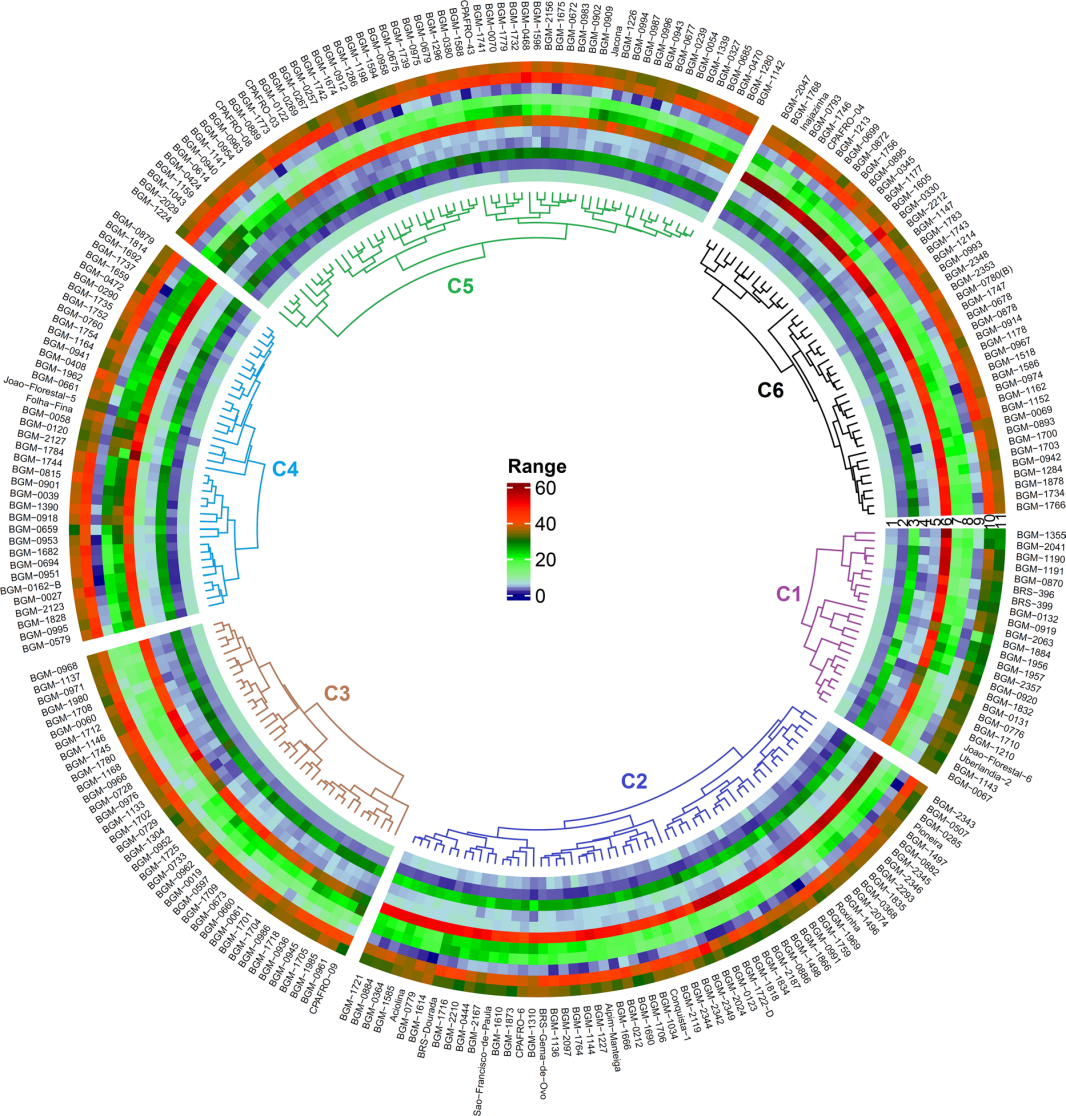
Circular heatmap of the biofortified cassava genotypes based on best linear unbiased prediction (BLUP), for several yield and root quality attributes. The characteristics are numbered from 1 to 11, in the following order: cyanogenic compounds content, pulp color of the roots, starch content, number of roots per plant, dry root yield, harvest index, shoot yield, fresh root yield, total carotenoid content, dry matter content by oven dry method and matter content by the gravimetric method

The BIC indicated the formation of six distinct groups of diversity based on yield and root quality traits (Fig. 4). There was a similar distribution of accessions in each cluster, that is, 24, 60, 38, 39, 60, and 44 genotypes in clusters 1, 2, 3, 4, 5, and 6, respectively. In cluster 1, 17 local cultivars and seven improved cultivars were allocated, including two biofortified improved cultivars (BRS Jari, BRS-396 and BRS-399). In cluster 2, 45 local cultivars and 15 improved cultivars were grouped, including BRS Dourada and BRS Gema de Ovo, which were also recommended as improved sweet cassava cultivars with high carotenoid content. Of the 38 genotypes in cluster 3, 11 were improved cultivars that in most cases were recommended for the Amazon region, while the remaining 27 local cultivars were collected in different regions of Brazil. In the case of cluster 4, only six of the 39 genotypes were classified as improved cultivars (many of them were obsolete), while more than 60% of the 33 local cultivars in this group were collected in the northeast region of Brazil. Cluster 5 had only six improved cultivars (with limited cultivation in Brazil) and 54 local cultivars, 20 of them originating from the north region, 23 from the northeast region of Brazil and the rest of Brazilian origin, but without precise identification of the collection region. Finally, seven of the 44 genotypes in cluster 6 were improved sweet cassava cultivars, while the other local cultivars had their origin in collection and traditions of use in the north (13 genotypes) and northeast (18 genotypes). The other local cultivars in cluster 6 had no defined origin.

**Fig. 5:**
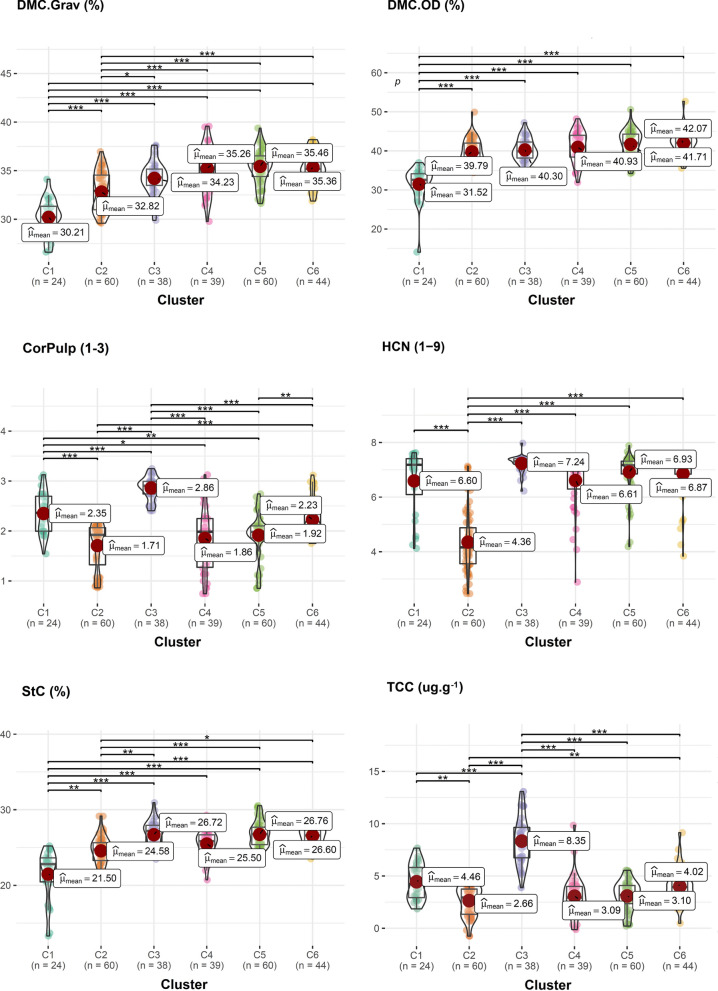
Box/Violin plot of best linear unbiased prediction (BLUP) of the different clusters of biofortified cassava genotypes based on root quality traits: DMC.Grav: dry matter content by gravimetric method, DMC.OD: dry matter content by oven dry method, PulpColor: pulp color of the roots, HCN: cyanogenic compounds content, StC: starch content, and TCC: total carotenoid content. *, **, and *** refer to significance of means comparisons by the Holm method at 0.05, 0.01, and 0.001, respectively

**Fig. 6:**
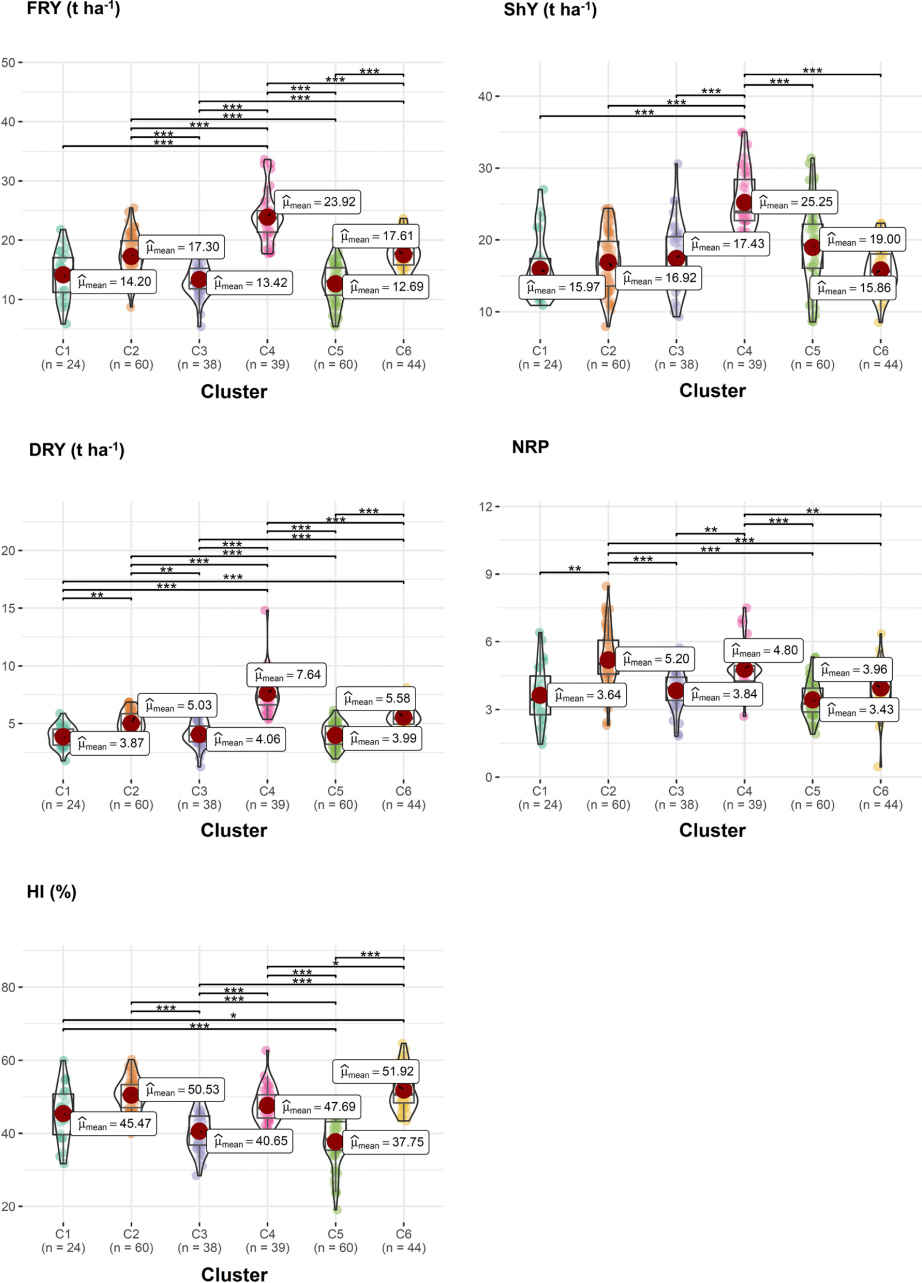
Box/Violin plot of the best linear unbiased prediction (BLUP) of the different clusters of biofortified cassava genotypes based on yield traits FRY: fresh root yield, ShY: shoot yield; DRY: dry root yield, NRP: number of roots and HI: harvest index. *, **, and *** refer to significance of means comparisons by the Holm method at 0.05, 0.01, and 0.001, respectively

The genotypes from cluster 1 exhibited low DMC in the roots (30.2% and 31.5% by the gravimetric and oven drying methods, respectively), intermediate levels of TCC (~ 4.5 µg g^− 1^) associated the average score of 2.35 for flesh color, indicating that most genotypes had cream-colored roots and intermediate HCN values ​​(6.6). Furthermore, the genotypes in this cluster had low StC (~ 21.5%) and low yield potential: FRY (~ 14.2 t ha^− 1^), ShY (~ 16.0 t ha^− 1^), PROD.Dry (~ 3.9 t ha^− 1^) and NRP (~ 3.6) (Figs. 5 and 6).

The genotypes in cluster 2 had low DMC, especially for the gravimetric method (32.8%), low TCC (~ 2.7 µg g^− 1^), and pulp colors (score ~ 1.7) indicating that although most genotypes had cream-colored flesh, some were very light cream. Further, cluster 2 had the lowest HCN score (4.3) and intermediate levels of StC (~ 24.5%) (Fig. 5). In terms of yield, median values ​​were identified for FRY (~ 17.3 t ha^− 1^), ShY (~ 17.0 t ha^− 1^) and DRY (~ 5.0 t ha^− 1^) and high HI (~ 50.5%) and NRP (~ 5.2) (Fig. 6).

Regarding the root quality traits, cluster 3 had intermediate DMC in the roots (34.23% and 40.30% by the gravimetric and oven dry methods, respectively), and the highest TCC (~ 8 0.3 µg g^− 1^) associated with the color of the pulp with an average score equal to 2.86 (most of the clones with yellow pulp) and high HCN (7.24). However, cluster 3 genotypes had low yield potential, especially for FRY (~ 13.4 t ha^− 1^), ShY (~ 17.4 t ha^− 1^), HI (~ 40.6%), DRY (~ 4.1 t ha^− 1^) and NRP (~ 3.8).

Similarly to cluster 2, cluster 4 had low TCC (~ 3.1 µg g^− 1^) and pulp color (score ~ 1.9), indicating that there were some clones with very light cream pulp, although most genotypes had cream pulp. However, cluster 4 had moderate StC (~ 25.5%) and HCN (6.6) values, as well as high DMC by both the gravimetric and oven dry methods (~ 35.3% and ~ 41.0%, respectively). Furthermore, cluster 4 was represented by the genotypes with the highest FRY (~ 24.0 t ha^− 1^), ShY (~ 25.3 t ha^− 1^), DRY (~ 7.6 t ha^− 1^) and NRP (~ 4.8).

The defining cluster 5 attributes were high DMC in the roots (35.5% and 41.7% by the gravimetric and oven dry methods, respectively), StC (~ 26.8%), and HCN (6.9). Further, carotenoid contents and root color were low (~ 3.1 µg g^− 1^ and 1.92, respectively). In agronomic terms, the genotypes in cluster 5 had low NRP (~ 3.4) associated with low FRY (~ 12.7 t ha^− 1^) and DRY (~ 3.9 t ha^− 1^), although they had high ShY (~ 19.0 t ha^− 1^) and consequently the lowest HI (~ 37.7%), when compared to the other clusters.

Finally, along with cluster 5, cluster 6 contained the highest DMC in the roots by the gravimetric (~ 35.3%) and oven dry (~ 42.1%) methods, as well as high scores in StC (~ 26, 6%) and HCN (6.9). The genotypes in cluster 6 had median TCC (~ 4.0 µg g^− 1^) and pulp color (2.2), indicating the presence of a mix of cream and yellow clones in this group. Another striking feature of this group was the presence of clones with high HI (~ 52%), as a result of the higher weight of FRY (~ 17.6 t ha^− 1^) compared to ShY (~ 15.9 t. ha^− 1^). The values ​​of DRY (~ 5.6 t ha^− 1^) and NRP (~ 4.0) were considered of moderate magnitude compared to the other groups.

### Genotype selection

The selection of the 30 best genotypes for recombination in the cassava breeding program has the potential to increase TCC by 27.05% when compared to the general germplasm average, reaching ~ 5.6 µg g^− 1^ (Table 3), associated with a strong reduction in HCN (-23%). In addition, there is the potential for significant gains for several agronomic attributes such as FRY (22.72%), ShY (14.08%), DRY (22.95%) and NRP (13.2%). However, the gains for DMC in the roots are potentially small (1.7% and 2.4% considering the gravimetric and drying methods, respectively).

**Table 3:** List of genotypes selected for recombination aiming at the generation of biofortified sweet cassava progenies based on the selection index, as well as the best linear unbiased predictors (BLUPs) plus the intercept of the germplasm and potential gains ($G$) of the selected clones

Genotype	Cluster	DMC.Grav	DMC.OD	TCC	FRY	ShY	HI	DRY	NRP	StC	PulpColor	HCN
Folha-Fina	4	35.91	37.84	3.32	32.86	31.47	47.96	9.79	4.91	25.03	2.07	2.89
BGM-1390	4	38.18	41.59	4.56	26.90	35.00	46.95	9.79	6.99	25.85	3.04	7.33
BGM-1962	4	35.30	39.15	9.85	24.31	23.77	47.32	7.78	4.09	25.54	2.68	7.40
BGM-0597	3	32.80	42.16	13.08	17.94	21.97	42.99	4.82	4.71	25.99	2.86	7.26
BGM-2127	4	33.40	36.44	3.26	32.90	18.61	62.69	14.81	4.75	21.97	1.14	5.49
CPAFRO-04	6	38.01	42.73	7.53	23.67	18.00	57.82	7.09	3.92	26.35	2.99	7.46
BGM-0019	3	36.05	44.10	11.67	17.15	20.47	42.90	5.30	4.26	28.87	2.74	7.23
BGM-1692	4	32.25	39.40	4.51	24.91	23.61	49.85	6.97	4.99	24.32	2.64	4.08
BGM-1700	6	34.81	39.98	9.14	20.06	20.00	49.04	6.46	4.21	25.89	1.91	6.15
BGM-1709	3	33.43	41.43	9.15	20.20	20.90	46.59	5.99	5.72	26.25	2.91	7.38
BGM-0290	4	38.06	43.36	2.57	23.95	23.03	50.40	7.74	3.42	24.70	2.98	4.45
BGM-1835	2	35.09	42.13	2.22	22.02	17.57	56.21	6.86	7.40	27.42	1.94	3.50
BGM-0368	2	33.35	38.18	3.56	21.17	16.55	53.80	5.92	6.11	26.04	2.09	2.46
BGM-2353	6	38.19	43.11	5.88	16.79	16.44	48.88	7.31	2.45	27.99	2.14	3.84
BGM-1745	3	37.51	42.74	8.96	16.08	14.21	50.83	5.51	3.62	29.10	2.94	7.40
BGM-0918	4	36.15	43.11	2.09	32.06	33.27	41.18	10.17	4.23	25.90	1.37	7.08
BGM-0444	2	32.44	37.42	4.61	23.25	22.20	51.45	6.46	3.50	23.93	2.07	3.95
BGM-0901	4	36.40	46.12	2.60	28.41	29.28	46.15	8.73	4.66	26.18	1.85	7.46
BGM-1780	3	37.62	41.61	8.98	15.85	14.44	49.04	5.34	4.20	27.38	3.18	7.40
BGM-0212	2	34.03	44.02	5.50	16.01	16.66	48.87	4.63	4.83	25.92	2.14	2.81
BGM-1814	4	34.47	41.05	3.66	22.15	24.35	48.90	6.51	4.62	28.93	2.00	4.83
BGM-0579	4	36.64	45.66	7.34	21.31	24.46	41.82	6.97	4.85	26.76	1.25	7.32
BRS Dourada	2	31.48	41.94	2.65	24.78	24.28	50.30	6.40	5.79	22.18	2.29	4.18
BGM-0120	4	31.40	34.11	2.07	33.62	34.84	50.70	8.90	5.19	22.67	1.80	7.32
BGM-0659	4	29.77	39.44	5.28	22.97	30.26	42.20	6.11	6.82	23.44	2.25	5.63
BGM-0760	4	32.22	38.93	4.09	25.79	18.32	54.34	7.18	4.72	24.70	2.25	5.63
BRS Gema de Ovo	2	35.75	42.73	3.67	17.90	20.99	46.57	5.61	5.74	25.77	2.31	3.08
BGM-0936	3	33.93	39.58	9.68	16.19	25.49	35.37	5.38	5.00	28.50	3.00	7.41
BGM-1497	2	34.87	35.46	3.56	20.74	16.32	57.14	6.55	3.66	23.25	2.09	2.56
BGM-2345	2	36.09	44.85	3.15	19.21	17.59	52.97	5.75	6.78	23.30	1.89	3.57
Mean of selected		34.85	41.01	5.61	22.71	22.48	49.04	7.09	4.87	25.67	2.29	5.48
General mean		34.22	39.99	4.09	16.49	18.57	45.40	5.06	4.15	25.36	2.11	6.80
$G$ (%)		1.67	2.44	27.05	22.72	14.08	6.61	22.95	13.18	1.21	7.93	-23.03

Dry matter content by gravimetric (DMC.Grav) and oven dry method (DMC.OD), total carotenoid content (TCC), fresh (FRY) and dry root yield (DRY), shoot yield (ShY), harvest index (HI), number of roots per plant (NRP), starch content (StC), cyanogenic compounds content (HCN) and pulp color of the roots (PulpColor).

Most of the genotypes selected for recombination belong to cluster 4 (43.3%) and cluster 2 (26.7%), whose average yield traits and low HCN were preponderant for the choice of individuals. However, genotypes from cluster 3 (20%) and cluster 6 (10%) with mainly high TCC and DMC were also selected.

## Discussion

### Variability of biofortified cassava germplasm

Combining nutritional quality with characteristics that define consumer acceptance, such as DMC and cyanogenic potential, has been one of the key concerns in the development of biofortified cassava cultivars. The increase in TCC in cassava roots and the factors involved in this process has been a constant focus of cassava breeding programs (Esuma et al. 2012; Ceballos et al. 2013, 2017; Rabbi et al. 2017; Beyene et al. 2017). Through recurrent selection, (Ceballos et al. 2013) increased the TCC from 10.3 to 24.3 µg g^− 1^ over a period of nine years. This increase is possible due to the predominance of additive genetic effects involved in TCC inheritance in cassava (Esuma et al. 2016) and that, therefore, population improvement can be effective in recombining favorable alleles and consequent increase in TCC. To achieve these goals in the breeding program, the exploration of the genetic variability present in the germplasm bank offers an initial approach for the identification of genotypes with high carotenoid values ​​combined with other traits of interest.

In this study, a screening of biofortified cassava germplasm from Brazil was conducted in order to evaluate the variability regarding the carotenoids content, yield and root quality-related traits. The genotypes were chosen because they had some level of yellow root pigmentation, given the high correlation between pulp color and TCC (Chávez et al. 2005; Sánchez et al. 2006; Esuma et al. 2016). The TCC values ​​of this study based on 265 genotypes showed greater amplitudes (0.075–13.08 µg g^− 1^) compared to the report by (Chávez et al. 2005) (1.02–10.40 µg g^− 1^) in 1789 genotypes of different origins and with different breeding levels. However, the range of variation of the DMC.Grav (10.72–57.23%) identified in 2022 genotypes was higher compared to the present study (26.61–39.57%), although the DMC.OD exhibited a very similar amplitude (14.07–52.67%). This amplitude for DMC.OD was superior to the findings of (Rabbi et al. 2017), which identified a variation of 8.4–45.4% based on 3232 genotypes developed at the International Institute of Tropical Agriculture (IITA).

The estimation of FRY values ​​is also of great importance for the ranking, selection, and adoption of cassava cultivars. In the present study, the general mean of 16.49 t ha^− 1^ of fresh roots was similar to that reported by Parkes et al. (2020) in elite parental yellow genotypes cassava from IITA (16.32 t ha^− 1^).

The genotypes evaluated in this study had different origins and levels of improvement (Supplementary Table 1), which is a likely cause of the wide genetic variability identified (Avijala et al. 2015). The evaluations of the most important agronomic and root quality traits allow the identification of parents with potential for the development of segregating progenies with maximum genetic variability and introgression of desirable genes available in germplasm (Oliveira et al. 2014).

### Genetic parameters and correlations between yield and root quality traits

The broad-sense heritability estimates were classified as low (${h}^{2}$ < 0.30), moderate (0.30 ≤ ${h}^{2}$≤ 0.60) and high (${h}^{2}$> 0.60) according to (Mehari et al. 2015). High estimates of broad-sense heritability were found for TCC (${h}^{2}$= 0.72). Similar ${h}^{2}$ values for TCC were reported in other studies, such as ${h}^{2}$= 0.73 (Esuma et al. 2012) and ${h}^{2}$ = 0.60 (Parkes et al. 2020). The absence of the genotype × environment interaction effect and high heritability for carotenoid content suggests the possibility of a good response to selection via recombination of favorable alleles. In addition, high heritability values ​​allow for more accurate parental selection (Parkes et al. 2020; Ceballos et al. 2017). However, the DMC gave${h}^{2}$ of medium magnitude (DMC.Grav 0.45 and DMC.OD 0.54), similar to the values ​​reported by (Parkes et al. 2020) and (Rabbi et al. 2017), who also found moderate ${h}^{2}$ values ​​(ranging from 0.40 to 0.51) for this trait.

The productivity traits FRY (${h}^{2}$ = 0.21), DRY (${h}^{2}$= 0.21) and ShY (${h}^{2}$ = 0.22) were of low heritability magnitude. These low values ​​tend to hinder the selection process for genetic improvement due to the greater influence of the environment (Ceballos et al. 2004). An alternative for improving traits with low heritability is the implementation of population improvement methods, such as recurrent phenotypic selection, in order to increase the frequency of favorable alleles along the selection cycles. The objective of such selection is to obtain a breeding population over different selection cycles by accumulating favorable alleles (Bos and Caligari 2007).

Correlations between TCC and HCN were positive, but of low magnitude. Therefore, it is possible to identify genotypes with high TCC content in the roots that can, at the same time, be used as sweet cultivars (low or intermediate HCN). Biofortified cassava cultivars should be used for “in natura” consumption because cooking the roots retains 95–100% of the carotenoid content, while processing the roots retains just 2–5% in the form of fufu (cooked cassava mass) and between 26 and 29% in the form of chikwangue (cassava mass soaked, rinsed, kneaded and steamed inside leaves, usually banana leaves) (Taleon et al. 2019). Therefore, it is necessary that the roots contain ≤ 100 mg kg^− 1^ of cyanogenic compounds (Peprah et al. 2020) to satisfy the acceptable limit of cyanide for human consumption (Nhassico et al. 2008; Falade and Akingbala 2010; Cliff et al. 2011).

The non-significant correlation between TCC × DMC (DMC.Grav and DMC.OD) in the biofortified cassava germplasm in Brazil allows the breeding of species to select individuals with high TCC and DMC simultaneously, to meet the cultivar of demand of end users. DMC in biofortified cultivars helps to retain carotenoids after cooking, preventing these pigments from flowing into water during the cooking process (Ceballos et al. 2012); therefore, cultivars that combine these two characteristics are more likely to be recommended for cultivation (Njukwe et al. 2013; Peprah et al. 2020).

Previous studies have shown that the TCC and DMC traits are independently inherited (Sánchez et al. 2006). In a more recent study (Sánchez et al. 2014), using germplasm samples from South America, the authors reported no correlation between TCC and DMC. However, the analysis of an African cassava germplasm panel (improved clones and local cultivars) demonstrated the existence of negative correlations between these characteristics in cassava roots, ​​ranging from − 0.22 to − 0.59 (Akinwale et al. 2010; Esuma et al. 2012; Njoku et al. 2015; Rabbi et al. 2017). The existence of this negative correlation in African cassava germplasm has been attributed to the presence of two loci on chromosome 1 at positions 24.1 and 30.5 Mbp, associated with cassava root color (highly correlated with carotenoid content) and a single locus for DMC in the region close to 24.1 Mbp on chromosome 1, causing a physical link between these two traits in African cassava germplasm (Rabbi et al. 2017).

Although the negative correlation between TCC × DMC is undesirable for the development of improved cultivars, it is possible to carry out successive recombinations in a recurrent selection scheme to break this physical linkage. Such an approach has been successfully used to obtain genotypes with high TCC value with acceptable DMC levels, raising the TCC over nine selection cycles by more than 100% compared to the population original (Ceballos et al. 2013; Sánchez et al. 2014).

In addition to the African germplasm, reports of the existence of a negative correlation between TCC × DMC have also been reported in transgenic cultivars, as the increase in TCC resulted in unwanted pleiotropic effects for reducing DMC (Failla et al. 2012; Beyene et al. 2017) reported a strong negative correlation between TCC × DMC in transgenic cassava roots grown in greenhouses (r = − 0.85, *p <* 0.001) and in field (r = − 0.92, *p* < 0.001) compared to non-transgenic controls. The main reason given by the authors for this negative correlation was the change in metabolic flux observed in the modified roots, in which the deviation of pyruvate for the carotenoids′ synthesis may have suppressed the ADP-glucose pyrophosphorylase activity (an important enzyme for starch biosynthesis).

The existence of non-transgenic native cassava germplasm in Brazil with zero correlation between TCC × DMC and, at the same time, with high heritability opens up important possibilities for recombining useful alleles for these two traits using simple breeding approaches, such as phenotypic selection. However, the use of more sophisticated approaches, such as genomic selection, has also shown high potential for the simultaneous rapid incorporation of useful alleles and amplification of these traits (Esuma et al. 2021). In particular, the authors of (Esuma et al. 2021) evaluated the genomic prediction capability within the baseline population for carotenoids and identified the possibility of predicting TCC and DMC in West African germplasm using this population, with mean accuracy of 0.40 and 0.34, respectively, using a G-BLUP predictive model.

### Selection of biofortified cassava genotypes

The selection of 30 genotypes for recombination in the cassava breeding program was based on high TCC and low HCN content, associated with high agronomic performance. Among these genotypes were some improved cultivars from Embrapa′s biofortification program (BRS Dourada and BRS Gema de Ovo), selected for their moderate TCC values ​​(3.16 µg g^− 1^) associated with high yield potential (21.34 t ha^− 1^ FRY, 42.33% DMC.OD and 6.0 t ha^− 1^ DRY). In addition, the BGM-0659, BGM-0212 and BGM-2353 genotypes exhibited TCC values ​​superior to the improved cultivars (5.55 µg g^− 1^), with good productive potential (18.59 t ha^− 1^ FRY, 42.19% DMC.OD, 6.01 t ha^− 1^ DRY), associated with low ​​(2.81 and 3.84 for genotypes BGM-0212 and BGM-2353, respectively) and intermediate HCN values (5.63 for the BGM-0659 genotype). These promising parents will compose the crossing blocks to generate progenies with high variability for these traits, taking care to ensure that the agronomic characteristics of the crop, such as root yield, are not negatively affected. This can be proven based on the potential genetic gain for yield attributes such as PROD.DRY (22.95%), FRY (22.72%) and ShY (14.1%). Therefore, this parent panel has a high potential to generate superior and transgressive progenies.

The primary importance of developing cultivars with high TCC is to nutritionally enrich the diet, from the supply of β-carotene as provitamin A, through its roots. In this sense, it is expected that in the next breeding cycles, the sweet cassava cultivars with high carotenoid content will be developed, and their agronomic and organoleptic characteristics adjusted, to achieve stability of cultivation in different environments.

## Final remarks

Wide genetic variability was identified in the biofortified cassava germplasm for attributes associated with root quality and agronomic performance, and the grouping of genotypes into six clearly distinct groups regarding the evaluated traits constitutes an important starting point for further conservation studies and use of these genetic resources for breeding purposes.

The magnitudes and directions of the correlations identified between the traits with the greatest agronomic impact on the development of sweet cassava cultivars will certainly contribute to the development of better selection strategies and composition of economic weights in selection indexes.

The higher heritability for TCC (${h}^{2}$ = 0.72) in cassava roots indicates that the selection of plants with this characteristic can be initiated at earlier stages of the breeding program in which there are non-replicated clones grown in only one environment, such as the seedling evaluation phase and clonal evaluation trial. However, for low or medium heritability traits such as DMC, selection should be initiated in trials that allow the evaluation with repetition within clones and in different environments, such as preliminary, advanced, and regional yield trials.

The 30 genotypes selected as parents for recombination in the biofortified sweet cassava program will allow the achievement of important and simultaneous genetic gains, especially for TCC (27.05%), DMC (2.44%), FRY (22.72%), DRY (22.95%) and HCN (-23.03%).
